# Sleep, physical activity and BMI in six to ten-year-old children measured by accelerometry: a cross-sectional study

**DOI:** 10.1186/1479-5868-10-82

**Published:** 2013-06-22

**Authors:** Mirjam Ekstedt, Gisela Nyberg, Michael Ingre, Örjan Ekblom, Claude Marcus

**Affiliations:** 1 Department of Clinical Science, Intervention and Technology, Division of Pediatrics, Karolinska Institutet, Stockholm, Sweden; 2 School of Technology and Health, KTH - Royal Institute of Technology, Stockholm, Sweden; 3 Department of Public Health Sciences, Karolinska Institutet, Stockholm, Sweden; 4 Åstrand Laboratory of Work Physiology, Swedish School of Sport and Health Sciences, Stockholm, Sweden; 5 Stress Research Institute Stockholm University, Stockholm, Sweden

**Keywords:** Pre-school children, Sleep, Physical activity, Sedentary, BMI, Accelerometry, Weekday, Weekend

## Abstract

**Background:**

The aim of this study is to describe the relationship between objective measures of sleep, physical activity and BMI in Swedish pre-adolescents. The day-to-day association between physical activity and sleep quality as well as week-day and weekend pattern of sleep is also described.

**Method:**

We conducted a cross sectional study consisted of a cohort of 1.231 children aged six to ten years within the Stockholm county area. Sleep and physical activity were measured by accelerometry during seven consecutive days. Outcome measures are total sleep time, sleep efficiency, sleep start and sleep end; physical activity intensity divided into: sedentary (<1.5 METS), light (1.5 to 3 METS) and moderate-to-vigorous (> 3 METS); and Body Mass Index standard deviations score, BMIsds.

**Results:**

Total sleep time decreased with increasing age, and was shorter in boys than girls on both weekdays and weekends. Late bedtime but consistent wake-up time during weekends made total sleep time shorter on weekends than on weekdays. Day-to-day *within*-subject analysis revealed that moderate-to-vigorous intense physical activity promoted an increased sleep efficiency the following night (CI < 0.001 to 0.047), while total sleep time was not affected (CI -0.003 to 0.043). Neither sleep duration (CI -0.024 to 0.022) nor sleep efficiency (CI -0.019 to 0.028) affected mean physical activity level the subsequent day. The *between*-subject analysis indicates that the sleep of children characterized by high moderate-to-vigorous physical activity during the day was frequently interrupted (SE = -.23, P < .01). A negative association between BMIsds and sleep duration was found (-.10, p < .01).

**Conclusions:**

Short sleep duration was associated with high BMI in six to ten year old children. This study underscores the importance of consistent bedtimes throughout the week for promoting sleep duration in preadolescents. Furthermore, this study suggests that a large proportion of intensive physical activity during the day might promote good sleep quality.

## Background

Inadequate sleep in early childhood is associated with multiple consequences such as attention difficulties, cognitive disruptions, poor school performance and mood disturbances [1]. In recent years, sleep curtailment has also gained attention as a potential contributor to the obesity epidemic in both adults and children [2-5]. Despite a growing body of evidence demonstrating an association between short sleep duration and current and future obesity in children aged 0–16 years [3,6-11], the causal relationship is still unclear. It is not known whether sleep loss affects the risk of obesity directly, through changes in function and levels of metabolic hormones such as glucagon-like peptide-1 [12] resulting in increased food intake, or if common behavioral or environmental factors such as hypo- or hyperactivity [13] and other types of stressors are involved, causing both sleep and weight disturbances [14,15]. However, few studies have included both assessments of physical activity intensity and sleep measures to clarify their reciprocal relationship and their association with BMI in children.

Despite limited scientific evidence [4], there is a widespread claim that children’s sleeping habits have changed dramatically over the last several decades, with decreased sleep duration compared to previous generations [5]. In fact, earlier studies on normal sleeping patterns among children in early childhood rely mainly on parental reports of sleep duration. Such reports might be unreliable and provide no information on sleep quality. Moreover, adults [16] and adolescents [17] seem more often to make up for a daily sleep deficit with longer weekend sleep, but the impact of sleep variability over the days of the week has rarely been studied among pre-adolescents.

Hypotheses invoking indirect behavioral pathways to link short or inadequate sleep and obesity propose that active play during the day is replaced by sedentary behavior such as TV watching due to short or fitful sleep [6,18,19]. Although both sedentary activity and sleep are activities with low energy expenditure, data suggest that increased time in sedentary activity and increased time spent sleeping have opposite effects on a child’s weight [18,19].

However, currently available information on the relation between daytime activity and sleep in pre-adolescents is scarce and contradictory. A large-scale study comprising 2,241 Estonian and Swedish youths did not suggest a link between sleep duration and physical activity [20]. In contrast, a Canadian study on 856 ten-to-twelve-year-old children showed that the relation between sleep and physical activity varied by day. Overall intensity of physical activity was higher among those maintaining recommended sleep (> 9 h) through weekdays and weekend, compared to those who slept the least (< 9 h) during the week and engaged in weekend “catch-up-sleep” [21]. Both studies used self-assessed or parental-reported sleep measures, which limits the interpretation of sleep duration and sleep quality. In two studies with objective measurements of both sleep and physical activity over one 24-hour period (n = 519) [22,23], higher levels of physical activity were associated with shorter sleep latency [23], but no associations were found between mean physical activity and sleep duration [22]. However, a single 24-hour period of actigraph measurement does not yield reliable estimates of sleep [24].

One cohort study of 275 eight-year-old children has objectively measured both sleep and daytime physical activity patterns for seven consecutive days [25]. This study indicated that physical activity and sleep were significantly related, such that a higher level of physical activity during the waking hours was associated with poorer sleep that night, and poorer sleep during the night was associated with a higher level of physical activity the following day. Given evidence of variability in children’s sleep on school days and weekends [21] and associations with obesity [26], there is rationale for exploring the weekday/weekend pattern of physical activity and sleep as well as the relation to obesity. Our study provides data on detailed day-to-day physical activity and sleep variables recorded by accelerometer over one week in a large cohort of children in middle childhood. We also have access to anthropometric values. Altogether, this allows us to a) describe sleep duration, sleep efficiency and sleep habits during weekdays and weekend, b) investigate how differences in physical activity are related to differences in sleep, after controlling for possible confounders such as age, gender, Body Mass Index, BMI, and weekday/weekend sleep, c) investigate individual day-to-day association between daytime physical activity and sleep, and d) describe the associations between sleep, BMI and the intensity of physical activity.

## Methods

This study forms part of a larger program, STOPP, the Stockholm Obesity Prevention Project [27]. The initial STOPP study was designed as a school-based intervention study over four school years (2001–2005).

### Settings and participants

A total of 1,538 children (age 6–10 years, grades 1–4) from ten schools within the Stockholm county area participated. The schools participating in the project had a mixed pupil population with children from both middle and working class families. The STOPP intervention aimed to increase the amount of physical activity by 30 minutes per child and day.

However, since the intervention did not result in any significant difference between groups in terms of physical activity [27], we considered pooling children in the intervention and control groups before analysis in the present study. Before that, we tested the potential influence of the intervention on sleep variables using a multilevel mixed ANOVA model with fixed effect estimates for intervention and weekday/weekend, including possible interactions (Table 1). The sleep variables indicated no effect of the intervention but that sleep was delayed, shortened and had reduced efficiency during weekends. The fact that no significant effect of the intervention was observed, despite the relatively large sample size and high statistical power of this study, suggested that any effect present in the data was likely to be very small. It was therefore judged to be safe to pool the children in the intervention and control groups before further analysis. The study was based on 1,231 children (610 boys and 621 girls) who fulfilled the inclusion criterion for physical activity, which was at least 600 minutes of activity registration per day for a minimum of four days including one weekend day. The study was approved by the Regional Ethical Review board in Stockholm.

**Table 1:** Results from mixed ANOVA model and marginal means for intervetion-by-weekend interaction

**Fixed effects**	**Sleep onset (hour)**		**Sleep end (hour)**		**TST (min)**		**Sleep efficiency (%)**	
	**chi2**	***p***	**chi2**	***p***	**chi2**	***p***	**chi2**	***p***
intervention	1.6	.210	1.3	.250	0.3	.565	0.0	.865
weekend	379	<.001	295.1	<.001	15.7	<.001	8.9	.003
intervention *weekend	0.0	.980	1.9	.742	0.1	.723	0.2	.686
**Random effects**	***est***	***SE***	***est***	***SE***	***est***	***SE***	***est***	***SE***
actiwatch (sd)	0.0	0,0	0,0	0.0	0.0	0.00	0.27	0.20
subject (sd)	0.52	0.02	0,36	0.02	0.38	0.01	3.32	0.08
ar1 (rho)	0.19	0.01	0,19	0.01	-0.04	0.01	0.11	0.02
residual (sd)	0.86	0.01	0,77	0.01	0.79	0.01	3.11	0.03
	**Sleep onset**		**Sleep end**		**TST**		**Sleep efficiency**	
**Marginal means**	***Mean***	***SE***	***Mean***	***SE***	***Mean***	***SE***	***Mean***	***SE***
control / weekdays	21.4	0.03	7.0	0.02	9.6	0.02	85.7	0.16
boys in weekend	21.8	0.03	7.3	0.03	9.5	0.03	85.4	0.17
girls on weekdays	21.5	0.03	7.0	0.02	9.6	0.02	85.7	0.16
girls in weekend	21.9	0.03	7.4	0.03	9.5	0.03	85.5	0.17

Abbreviations: *chi2* chi-square test statistic, *p* p-value, *mean* estimated marginal mean of groups, *SE* standard error of estimated marginal mean, *sd* standard deviation of random effect, *rho* autoregressive correlation of residuals. Note: weekend is defined as Friday and Saturday, weekdays are Sunday-Thursday. n = 1,160 individuals and 7,164 observations of sleep data.

### Main outcome measures

Physical activity, PA, was assessed objectively using wrist-worn accelerometers (Actiwatch^®^ [AW], model 4, Cambridge Neurotechnology Ltd., Cambridge, UK). Every school week, twenty children (ten from intervention and ten from control schools) were randomly selected for accelerometry assessments during seven consecutive days. The children were instructed to wear the AW around the clock for the whole week except while swimming or bathing.

PA was calculated as total counts divided by the total recording time and expressed as counts per minute (cpm). Mean PA (between 8 AM and 9 PM) was calculated over the seven-day period. The percentages of time (minutes/day) spent at three different intensities of physical activity were used as outcome variables. The activities were initially defined as sedentary (below 1.5 METS), light (between 1.5 and 3 METS), moderate (between 3 and 6 METS) and vigorous (above 6 METS). The thresholds for the intensities of activity were defined as follows: sedentary below 332 cpm, light between 332 and 1004 cpm, moderate between 1004 and 2336 cpm, and vigorous above 2336 cpm. The moderate and vigorous categories were combined into one: moderate-to-vigorous physical activity (MVPA). Sedentary activities may include sitting or playing computer games, while the lower range of the light activity corresponds to slow walk (between 2.3 and 3.0 km h^-1^) (unpublished data). Sedentary activity after 9 PM was separated from sleep by the algorithm used to identify sleep. Data were excluded from the analyses if sequences during the waking hours indicated ten or more consecutive minutes of zero counts [28], suggesting that the monitor had been removed from the body. The epoch length was set to one minute.

Sleep was assessed using the same device as for the physical activity assessment. In normal populations of children, wrist actigraphy has reasonable reliability and validity in sleep-wake detection compared with laboratory-based polysomnography [29]. Sleep data were analyzed with Actiwatch Activity & Sleep Analysis 5 version 5.32 software (Cambridge Neurotechnology Ltd), which identified each epoch as sleep or waking using the mathematical model validated by Cole et al. [30]. Since there were no logs to indicate “lights off”, or sleep end, sleep onset was manually set as the first epoch scored as immobile that was sustained for ten minutes or longer. Sleep end was set as the last epoch scored as immobile that was sustained for ten minutes or longer. Sleep latency, the interval between bedtime and sleep onset, was not estimated. Within the manually set sleep period time, the following sleep measures were analyzed: total sleep time, TST, i.e. the sum (in minutes) of all sleep epochs between sleep onset and sleep end; and sleep efficiency, SE, i.e. the percentage of sleep per time in bed.

The children’s weight and height were measured by two trained research assistants using standard clinical procedures with a digital scale (Tanita BWB 800S, Tanita, Tokyo, Japan) and a Harpenden Stadiometer. Weight was measured to the nearest 0.1 kg and height to the nearest 0.1 cm. BMI was calculated as weight (kg) divided by height (m) squared. Body Mass Index standard deviations score, BMIsds, was calculated according to Rolland-Cachera [31]. The prevalence of overweight and obesity was 21.6%, calculated according to Cole et al. [32].

### Statistical analysis

Data were analyzed by means of multilevel mixed ANOVA models with fixed effect estimates for age group, sex and weekend, including all possible interactions. The model was fitted independently to each activity/sleep variable reported in the present study. A total of 29 individual Actiwatch monitors were used to collect data for the 1,231 subjects during 8,252 days. The data were modeled with a three-level design (day, subject and Actiwatch monitor), using random effects to account for variation between Actiwatch monitors and subjects. In addition, an autoregressive covariance structure was used to model residual error across days of data collection.

Between-subject associations of sleep and activity were studied using model-based empirical Bayes estimates (also known as best linear unbiased predictors, BLUP) from the main ANOVA model presented above. These estimates were adjusted for all fixed and random effects to provide estimates of subject-specific deviation from the group independent of age, sex (and all interactions) as well as variation between Actiwatch monitors. Correlations between empirical Bayes estimates were reported in a standard correlation matrix.

In addition, we used the main ANOVA model to estimate within-subject (day to day) covariation between daytime activity and sleep by means of systematically adding a single (repeated measures) fixed effect covariate (of a sleep/activity variable) to the ANOVA model described above and estimate its effect. Sleep and activity data were standardized within-subject for easier interpretation of the results.

## Results

### Sleep pattern in adolescent girls and boys during weekdays and weekend

All sleep variables were related to age, suggesting later sleep onset and later wake-up, shorter TST and greater SE with increased age (Table 2). A significant main effect of sex for sleep onset and TST was found. Girls went to sleep earlier and had a longer TST than boys at all ages. There were no differences between boys and girls in SE and sleep end.

**Table 2:** Results from the mixed ANOVA-model and marginal means for age-by-gender-by-weekend interaction

**Fixed effects**		**Sleep onset**		**Sleep end**		**TST**		**Sleep efficiency**	
		**chi2**	***p***	**chi2**	***p***	**chi2**	***p***	**chi2**	***p***
sex		5.2	.022	1.2	*.267*	21.0	<.001	0.3	.572
age		122.2	<.001	11.3	*.010*	126.1	<.001	20.9	<.001
weekend		353.2	<.001	295.1	*<.001*	18.0	<.001	7.1	.008
sex*age		1.5	.677	1.9	.595	0.7	.883	1.3	.725
sex*weekend		0.9	.350	2.1	.146	0.3	.579	3.2	.072
age*weekend		5.9	.117	6.9	*.*074	7.9	*.048*	3.3	.352
sex*age*weekend		5.4	.144	1.6	*.*651	6.2	.101	2.4	.497
**Random effects**		***est***	***SE***	***est***	***SE***	***est***	***SE***	***est***	***SE***
actiwatch (sd)		0.009	0.00	0.0	0.00	0.0	0.00	0.31	0.20
subject (sd)		0.490	0.02	0.36	0.02	0.34	0.01	3.29	0.08
as1 (rho)		0.19	0.01	0.19	0.01	-0.04	0.01	0.11	0.02
Residual (sd)		0.86	0.01	0.77	0.01	0.79	0.01	3.11	0.03
		**Sleep onset**		**Sleep end**		**TST**		**Sleep efficiency**	
**Marginal means**	***Age***	***Mean***	***se***	***Mean***	***SE***	***Mean***	***SE***	***Mean***	***SE***
Boys on weekdays	6	21.3	0.06	7.0	0.05	9.8	0.05	84.7	0.35
	7	21.3	0.05	7.0	0.04	9.6	0.04	85.3	0.28
	8	21.6	0.06	7.0	0.05	9.5	0.04	85.7	0.33
	9	21.7	0.05	7.1	0.04	9.3	0.04	86.3	0.27
Boys in weekend	6	21.5	0.08	7.2	0.07	9.7	0.06	84.6	0.39
	7	21.7	0.06	7.2	0.05	9.5	0.05	85.2	0.31
	8	22.1	0.07	7.3	0.06	9.2	0.06	85.8	0.36
	9	22.1	0.06	7.5	0.05	9.3	0.05	86.1	0.30
Girls on weekdays	6	21.1	0.06	7.0	0.05	9.9	0.04	85.3	0.33
	7	21.3	0.05	7.0	0.04	9.7	0.04	85.6	0.29
	8	21.5	0.05	7.0	0.04	9.5	0.04	85.8	0.31
	9	21.6	0.05	7.1	0.04	9.4	0.04	86.3	0.28
Girls in weekend	6	21.5	0.07	7.3	0.06	9.8	0.06	85.1	0.37
	7	21.7	0.06	7.3	0.05	9.6	0.05	84.9	0.32
	8	21.9	0.07	7.4	0.06	9.5	0.05	85.7	0.34
	9	22.0	0.06	7.5	0.05	9.4	0.05	85.9	0.31

Abbreviations: *chi2* chi-square test statistic, *p* p-value, *mean* estimated marginal mean of groups, *SE* standard error of estimated marginal mean, *sd* standard deviation of random effect, *rho* autoregressive correlation of residuals. Note: weekend is defined as Friday and Saturday, weekdays are Sunday-Thursday. n = 1,160 individuals and 7,164 observations of sleep data.

A significant difference between weekday and weekend nights was shown in all sleep variables. Sleep patterns were delayed on weekends compared with weekdays, with sleep onset markedly more delayed than sleep end. A shorter TST and a lower SE was found on weekends than on weekdays. No interaction effect was found except for TST, age and weekend, suggesting a shorter weekend sleep with increased age. Sleep pattern and SE varied similarly across age and day of the week in both boys and girls.

### Between-subject correlations between sleep variables, physical activity and BMI

Between-subject correlations reported in Table 3 showed a strong correlation between TST and sleep onset (r = -.60) and a markedly weaker relation between TST and sleep end (r = .16). A positive correlation between weekend and TST (r = .46) and a negative correlation with sleep onset (r = -.26) indicate that children with less tendency to shorten their sleep by going to bed later during weekend nights were also likely to sleep longer.

**Table 3:** Between-subjects correlations of stable individual differences (BLUP)

	**BMI**	**BMI sds**	**Obese vs normal weight**	**Overweight vs normal weight**	**Mean activity**	**Time spent sedentary**	**Time spent in MVPA**	**Sleep onset (time of day)**	**Sleep end (time of day)**	**Sleep efficiency**	**TST**
Total sleep time, TST (min)	**-.085**	**-.099**	-.042	-.039	-.068	.051	-.052	**-.596**	**.159**	**-.158**	**1**
Sleep efficiency, SE (%)	.024	-.029	.038	.019	**-.233**	.199	**-.226**	**.139**	.024	**1**	
Sleep end (time of day)	-.064	-.060	-.051	-.043	-.085	.109	**-.081**	**.692**	**1**		
Sleep onset (time of day)	.005	.018	-.009	-.014	-.025	.056	-.032	**1**			
Time spent in MVPA (min)	**-.124**	**-.102**	**-.109**	-.108	**.976**	**-.837**	**1**				
Time spent sedentary (%)	.052	.044	.052	.046	**-.847**	**1**					
Mean activity (cpm)	**-.115**	**-.096**	**-.100**	**-.107**	**1**						
Owerweight vs normal weight	**.765**	**.710**	**.477**	**1**							
Obese vs normal weight	**.644**	.**569**	**1**								
BMIsds	**.914**	**1**									
BMI	**1**										

Abbreviations: *BLUP* best linear unbiased predictor, *TST* total sleep time; *SE* sleep efficiency, *WASO* wake after sleep onset, *MVPA* moderate-to-vigorous physical activity, *cpm* counts per minute, *BMIsds* Body Mass Index standard deviations score, *BMI* body mass index. Significant correlations (p < .01) are in bold text. n = 1,160 individuals and 7,164 observations of sleep data.

The between-subject analyses further revealed that low SE was related to more time in MVPA (r = -.23). The same relationship was found for the mean PA (SE r = -.23), suggesting that both children who were frequently active during daytime and children with a high intensity of PA during daytime had a poorer sleep efficiency (i.e. more fragmented sleep). Further, a positive correlation between SE and sedentary activity (r = .20) was found. No significant correlation was found between TST and PA variables.

A negative correlation between TST and BMIsds (r = -.10) indicates that short TST was related to increased BMI. The effect size was weak, however, and no significant correlation was found with other sleep variables. A similar effect of short time in MVPA and high BMIsds (r = -.10) was found, suggesting a relation between low PA and high BMIsds.

### Day-to-day co-variation between sleep and physical activity within individuals

The within-subject (day-to-day) co-variation between sleep and PA (Table 4), showed that both long time spent in MVPA and high mean PA during the day predicted high SE the following night, while long time spent in sedentary activity predicted low SE the following night. However, time spent in sedentary activity, MVPA and mean PA did not affect TST the following night. In contrast, the temporal association between PA and sleep was not reciprocal, meaning that TST and SE did not predict MVPA, sedentary behavior or mean PA the following day. Late sleep onset and sleep end (i.e. eveningness) predicted high mean PA and long time spent in MVPA, whereas early sleep onset and early sleep end (morningness) predicted long time spent in sedentary activity the following day. All values were controlled for gender, age, day of the week and all interactions.

**Table 4:** **The day-to-day analysis of sleep and physical activity *within *individuals**

**Dependent variable**	**Predicting covariate**	**Coef.**^**a**^	**95% CI**
**A. Physical Activity**			
Total activity (cpm)	Sleep efficiency	0.005	-0.019 to 0.028
	Total Sleep time	-0.001	-0.024 to 0.022
	Sleep end	**-0.059**	**-0.087 to -0.031**
	Sleep start	**-0.046**	**-0.074 to -0.017**
			
MVPA (minutes)	Sleep efficiency	<0.001	-0.023 to 0.025
	Total sleep time	0.008	-0.0156 to 0.032
	Sleep end	**-0.050**	**-0.078 to -0.022**
	Sleep start	**-0.048**	**-0.077 to -0.020**
			
Sedentary (%)	Sleep efficiency	-0.004	-0.029 to 0.020
	Total sleep time	0.004	-0.020 to 0.027
	Sleep end	**0.055**	**0.027 to 0.083**
	Sleep start	**0.039**	**0.009 to 0.067**
**B. Sleep**			
Sleep efficiency (%)	MVPA	**0.024**	**<0.001 to 0.047**
	Sedentary	**-0.038**	**-0.061 to -0.014**
	Total activity	**0.024**	**<0.001 to 0.048**
			
Sleep onset (hour)	MVPA	**0.082**	**0.059 to 0.105**
	Sedentary	**-0.108**	**-0.131 to -0.085**
	Total activity	**0.099**	**0.077 to 0.126**
			
Sleep end (hour)	MVPA	**0.080**	**0.057 to 0.103**
	Sedentary	**-0.115**	**-0.138 to -0.093**
	Total activity	**0.094**	**0.071 to 0.116**
			
Total sleep time (minutes)	MVPA	0.020	-0.003 to 0.043
	Sedentary	-0.003	-0.026 to 0.021
	Total activity	0.016	-0.007 to 0.039

^a^Values are standardized coefficients and their 95% confidence interval, CI. Values are controlled for gender, age, weekend/weekday and all interactions. Upper part (A) showing the effect of sleep on the following day’s activity. Lower part (B showing the effect of activity on the following night’s sleep. Variables with a confidence interval, CI, not including “zero” are in bold text. Abbreviations: *cpm* counts per minute, *MVPA*  percent of the recorded time spent in MVPA, *Sedentary* percent of the recorded time spent in sedentary activity, *Total activity* mean activity (cpm), *n* 1,160 individuals and 7,164 observations of sleep data.

Mean activity level was lower during weekends compared with weekdays (698 vs. 810 cpm, p < 0.001). However, the day of the week variable was not a significant predictor of sleep outcomes, and accordingly separate analyses for weekend day and weekdays were not carried out. Descriptive data on PA variables are presented in detail elsewhere [33].

## Comment

### Sleep duration and sleep pattern on weekdays and weekends

Mean TST decreased with increasing age on both weekdays and weekends. The main reason for the age-dependent decrease in TST was a later sleep onset, whereas wake-up times are less related to age, as shown previously [34]. TST was markedly shorter in the present study compared with age-specific normative reference values for sleep duration [35]. Scientific evidence for the widespread notion of a general decline in sleep duration over the past decades is scarce and contradictory [4,35-38]. However, most earlier studies are based on self-reported data, and this might explain the variability in sleep duration, since both night awakenings [39] and sleep time are underreported [29]. An increasing number of studies using objective measures of sleep are reporting less than the recommended 9 hours of sleep in children, though [25,26,40]. This is an alarming trend, considering the long-term consequences of sleep deprivation on health and performance at school. Good sleep practices are developed within the family culture and associated with better sleep throughout life [41], which underlines the importance of parental involvement in modeling healthy sleeping habits from an early age. Girls went to sleep earlier and slept longer than boys on both weekdays and weekends, which is consistent with other findings [36,42]. It is still unknown whether young women have a greater physiological need for sleep than men, or if the difference is due to social-cultural influences. However, there is some support for such socio-cultural differences between boys and girls that may affect sleep. Adolescent boys are more likely than adolescent girls to have a television or computer game console in their bedroom. On the other hand, children who attend at least one extracurricular activity do not show any gender differences in self-reported sleep [34,36].

TST was *shorter* on weekends than on weekdays due to later sleep onset without a sufficient compensatory delay in wake-up time. This confirms a previous study of seven-year-old children [22]. The mean duration of sleep as defined by Actiwatch in that community cohort was 50 minutes shorter than the parental reported sleep. In most studies based on parental reported sleep, no difference between weekend and weekday sleep in children is reported, probably due to the fact that six- to nine-year-old children are not directly observed by their parents after bedtime. In our study, a tendency towards a later wake-up hour on weekends was seen at age nine, which is consistent with the sleep phase shift to later hours on weekends which is seen in adolescents [17].

The consequences of a shorter weekend sleep on physical activity are not well explored, however. The study by Stone et al. [21] suggests that weekday-weekend regularity matters, since ten- to twelve-year-old children who maintained the recommended sleep regularity across the days of the week showed the most positive activity profiles. We did not find any significant correlation between weekend sleep and BMIsds or PA in our study. One reason may be that we have studied relatively young children, with no tendency to “catch up sleep” in weekends and no concomitant increase of the time in sedentary activity.

Taken together, the overall short sleep pattern described in this study with a lack of recuperation sleep in weekends demonstrates that regular bedtime is an important determinant of sleep duration in children and pre-adolescents. Considering that unhealthy sleeping patterns increase the risk of sleep deprivation in children as well as when transitioning to adolescence [43], the benefit of regular sleeping hours throughout the week is clearly evident. However, possible short- and long-term consequences of short sleep, in terms of physical activity as well as obesity development have to be explored in longitudinal studies.

### Sleep and physical-activity variation between and within individuals

The *between-subject analysis* indicates that a high proportion of intense PA during the day was associated with frequently interrupted sleep, and that sedentary activity was positively related with SE. This is an unexpected finding but in line with a recent correlational study on American children (n = 55) in the same age group, describing an inverse correlation between the hours of sleep per night and the number of moderate-vigorous activity bouts [40]. A possible explanation may be that some children have generalized physiological hyperarousal and stress [44], which in turn may be due both to environmental and to hereditary factors. Correlations in childhood between high hyperactivity scores and turning during sleep [45], disrupted sleep [46] or neuro-developmental problems have previously been detected [47]. The mechanisms involved are unknown, however, and it is not known whether fragmented sleep patterns among physically more active children actually reflect poor sleep, or if an inherent high activity level in itself may be manifested as a higher activity level during both day and night (i.e. more turning around and movements during sleep).

Our study design also allowed us to clarify whether PA and sleep are reciprocally related. Contrary to the between-individual analysis discussed above, the day-to-day analysis of sleep and physical activity *within* individuals showed that a high proportion of MVPA (and a low proportion of sedentary activity) during the day promoted sleep efficiency during the following night, but did not affect sleep duration. In contrast, neither sleep duration nor sleep efficiency affected physical activity level the subsequent day, suggesting that daytime PA may be more dependent on motivation and scheduled time for activity than on sleep per se.

These associations between high daytime PA and improved sleep the following night are supported by experimental evidence in adults as well as children [48]. The study of twelve-year-old children by Dworak et al. [48] showed that high-intensity exercise resulted in improved slow-wave sleep the following night, but did not affect sleep duration. That study tested the effect of exhaustive endurance exercise on polysomnographically measured sleep, while our study measured “free-living activities” during a week, making comparisons difficult. However, our findings indicate that also time spent in unscheduled moderate-to-high intensity activity during the day may promote good sleep efficiency the subsequent night. Sleep duration in contrast, might be more sensitive to environmental factors like school scheduling, TV-watching and other social activities, than the factual need for sleep, which might explain why sleep duration was not affected.

Only a few earlier studies have tested the day-to day relation between objectively measured sleep and unstructured PA in children, and the results are variable. Pesonen et al. [25] found, in contrast to our study, a bidirectional relationship between high level of PA and poor sleep, while Nixon et al. [22] reported no significant association between sleep duration and PA, as measured for 24 hours. However, these findings have to be interpreted with caution until they are confirmed/refuted by further studies.

### Sleep and obesity

The negative association between BMI and sleep duration in this study is in concert with evidence from a growing number of cross-sectional and longitudinal studies that short sleep duration is associated with high probability of being overweight [2,3,6,22,49]. The effect size was small, however. Differences between effect estimates in different studies could indicate that factors linked to cultural habits, including behavioral differences, might mediate the association between sleep duration and overweight. A large European study including 7,867 children from eight countries showed that regional differences might act as effect modifiers of the association between sleep duration and overweight [49]. Our data showed a negative correlation between BMI and MVPA, which could represent such a mediating factor. Another possible explanation of the small effects sizes is that children may have been rather restricted concerning bed time and wake up time during regular school weeks.

### Strengths and limitations

The strengths of our study are the large sample size and the fact that we assessed sleep and physical activity with objective measures using seven consecutive 24-h recordings, which allows us to examine potential correlations between physical activity and sleep both weekdays and weekends. A weakness of this study is the lack of sleep diaries, which makes assessment of sleep times more difficult. In addition, cultural and environmental characteristics seem to modulate individual determinants of sleep in children [50], making general conclusions hazardous. The effects found in the present study may be regarded as rather limited. However, the participating children were measured during a normal school week, which means that their sleep and physical activity may have been restricted by parents and school routines. Therefore, the associations between physical activity and sleep variables may have been underestimated. Finally, it should be emphasized that temporal associations do not prove a cause-effect relationship. The associations in our study may also be dependent on additional factors that are not specified here.

In conclusion, this study adds to the growing body of evidence demonstrating that short sleep is associated with risk of increased BMI even in children, and it underscores the importance of consistent bedtimes throughout the week for promoting sleep duration in this age group. Furthermore, this study suggests that a large proportion of intensive physical activity during the day might promote good sleep quality. It is important to recognize that current recommendations on physical activity are based on epidemiological associations between self-reported physical activity and health outcomes. Given the discrepancies between self-reported and objective measures in children, evidence-based research grounded in objective measurements of physical activity and sleep are needed. This may improve development of guidelines and recommendations for activity levels and sleep that can be tested in longitudinal studies.

## Consent

Written informed consent was obtained from the patient’s guardian/parent/next of kin for the publication of this report and any accompanying images.

## Competing interest

The authors declare that they have no conflict of interest.

## Authors’ contributions

CM is the director of the research group, ME was the leading author, GN was responsible for data collection. All authors have contributed to the draft with their own expertise. MI carried out the statistical analysis, GN and ÖE have provided their expertise in physical activity, CM in obesity, endocrinology and pediatrics and ME in sleep physiology and sleep measures. All authors read and approved the final manuscript.
